# The Involvement of Calcium Channels in the Endoplasmic Reticulum Membrane in Nonalcoholic Fatty Liver Disease Pathogenesis

**DOI:** 10.7759/cureus.49150

**Published:** 2023-11-20

**Authors:** Sarah S Albalawi, Ahmed Aljabri, Mohannad Alshibani, Mohammed M Al-Gayyar

**Affiliations:** 1 PharmD Program, Faculty of Pharmacy, University of Tabuk, Tabuk, SAU; 2 Pharmacy Practice, Faculty of Pharmacy, King Abdulaziz University, Jeddah, SAU; 3 Pharmacy Practice, Faculty of Pharmacy, University of Tabuk, Tabuk, SAU; 4 Pharmaceutical Chemistry, Faculty of Pharmacy, University of Tabuk, Tabuk, SAU; 5 Biochemistry, Faculty of Pharmacy, Mansoura University, Mansoura, EGY

**Keywords:** mitochondria, endoplasmic reticulum, non-alcoholic fatty liver disease (nafld), inositol trisphosphate receptor (ip3r), calcium ions

## Abstract

Nonalcoholic fatty liver disease (NAFLD) is a prevalent and complex condition that affects millions of people globally. It occurs when fat, primarily triglycerides, accumulates in liver cells, leading to inflammation and damage. Calcium, an essential mineral, is involved in various physiological processes, including the regeneration process following liver injury. The endoplasmic reticulum (ER), a complex organelle involved in protein synthesis and lipid metabolism, regulates intracellular calcium levels. Dysregulation of this process can lead to calcium overload, oxidative stress, and cellular damage, all of which are hallmarks of NAFLD. Inositol 1,4,5-trisphosphate receptor (IP3R), a type of calcium ion channel, is found throughout the body, including the liver. IP3R is classified into three subtypes: IP3R1, IP3R2, and IP3R3, and it plays a critical role in regulating intracellular calcium levels. However, excessive calcium accumulation in the mitochondria due to an overload of calcium ions or increased IP3R activity can lead to NAFLD. Therefore, targeting calcium channels in the ER membrane may represent a promising therapeutic strategy for preventing and treating this increasingly prevalent metabolic disorder. It may help prevent mitochondrial calcium accumulation and reduce the risk of hepatic damage. This review article aimed to review the relationship between IP3R modulation and the pathogenicity of NAFLD, providing valuable insights to help researchers develop more effective treatments for the condition.

## Introduction and background

Nonalcoholic fatty liver disease (NAFLD) is a chronic condition where fat, predominantly triglycerides, accumulates in liver cells. NAFLD is a common condition and is associated with insulin resistance, and its severity ranges from asymptomatic stages to cirrhosis. Studies show that 50% of diabetes patients and 76% of obese individuals also have NAFLD, revealing that diabetes and obesity are significant risk factors along with hyperlipidemia and genetic factors [1]. Recent statistics have revealed that 10-40% of individuals worldwide suffer from NAFLD, with a prevalence of 16.6% in Saudi Arabia, according to the latest study. This number is expected to increase with the rise of diabetes and obesity [2].

NAFLD has become a major concern for healthcare systems due to its possible progression to advanced liver disease and hepatocellular cancer, which can lead to significant direct and indirect cost burdens [3]. NAFLD diagnosis involves various invasive and noninvasive methods. The invasive method involves a biopsy examination. The noninvasive methods include systems such as Fatty Liver Index (FLI), NAFLD Liver Fat Score (NLFS), Lipid Accumulation Product (LAP), and imaging for Hepatic Steatosis [4].

Pharmaceutical companies have made extensive efforts to develop drugs for treating NAFLD, but none have been approved so far. This is partly due to the fact that the underlying causes of NAFLD are not fully understood, and there is no single drug that can address all the different factors that contribute to the condition. To effectively manage NAFLD, weight reduction and pharmacotherapy are recommended. Currently, the only effective therapy for NAFLD is behavior modification, which focuses on promoting a healthy lifestyle. This includes a healthy diet low in calories, saturated fats, and added sugars, as well as regular physical activity. Exercise is particularly important for reducing liver fat, improving insulin resistance, and reducing inflammation [5].

In addition to lifestyle changes, there are also some medications and supplements that may help treat NAFLD. These drugs include pioglitazone, which improves hepatic steatosis, necroinflammation, and fibrosis; glucagon-like peptide 1 (GLP-1) agonists that improve hepatic steatosis and necroinflammation; and sodium-glucose cotransporter 2 (SGLT2) inhibitors that improve hepatic steatosis, necroinflammation, and liver enzymes. However, these drugs should only be used under the guidance of a healthcare provider as they may lead to adverse effects such as bone loss, fluid retention, acute kidney injury, euglycemic diabetic ketoacidosis, and genitourinary infection [4]. Moreover, these medications should not be seen as a substitute for lifestyle changes. Overall, a comprehensive approach that combines healthy behaviors, medications, and supplements tailored to the individual needs of the patient is the best way to manage NAFLD and prevent its progression to more serious liver disease [6]. However, unfortunately, patients’ adherence to behavior programs remains low [1].

## Review

Physiological role of calcium in the liver

Calcium ions play a crucial role in various physiological processes in the liver, acting as a second messenger and influencing bile secretion, liver cell regeneration, glucose and lipid metabolism, and cell proliferation. Furthermore, calcium is essential for maintaining liver homeostasis and supports the regeneration process following acute or chronic injury [7]. Aberrant calcium signals have been linked to various liver pathologies such as primary biliary cirrhosis, cholestasis, alcoholic liver disease, and NAFLD due to the significance of calcium-sensitive processes [8].

Maintaining the balance of calcium signals between the nucleus, cytoplasm, and mitochondria is of utmost importance in liver regeneration following injury. The expression and distribution of calcium channels in different organelles are also critical in this process [9]. The changes in intracellular calcium concentration are influenced by the influx of extracellular calcium and the release of calcium from intracellular calcium stores such as the endoplasmic reticulum (ER), mitochondria, and lysosomes. When external factors stimulate cells, various calcium channels are opened or closed, leading to impaired calcium homeostasis. This, in turn, can cause a series of metabolic damage and liver regeneration disorders, including NAFLD [10]. Thus, maintaining the delicate balance of calcium signaling within the cells of the liver is critical for the proper functioning of the organ and to prevent the onset of various liver disorders.

In NAFLD, the calcium signaling pathway may become dysregulated, leading to various adverse outcomes. For instance, alterations in cystic calcium levels can result in calcium depletion in ER, activating the unfolded protein response (UPR) and causing insulin resistance. In drug-induced liver injury (DILI), ER stress can be triggered by different mechanisms that cause calcium imbalance. N-acetyl-p-benzoquinone imine (NAPQI), a highly hepatotoxic substance, binds to glutathione-S-transferases and other proteins, leading to endoplasmic reticular stress [11]. Calcium signaling disruption in the liver can result in insulin resistance, and ER-mitochondria miscommunication contributes to insulin resistance, which is considered a high-risk factor for NAFLD [12]. Therefore, it is critical to maintain a healthy calcium signaling pathway in the liver to prevent adverse outcomes and promote overall liver health.

ER stress and NAFLD

ER is a complex organelle responsible for various cellular processes, including the synthesis, folding, and modification of secretion and transmembrane proteins. Additionally, it serves as a storage site for calcium and lipids and plays a key role in their detoxification and synthesis. Despite its many functions, ER is vulnerable to various internal and external factors that can disrupt its delicate balance and lead to ER stress [13].

ER stress occurs when the ER's homeostatic balance is disturbed due to a buildup of unfolded or misfolded proteins, calcium depletion, and lipid synthesis disorders. This can occur due to factors such as glucose deficiency, exposure to toxins, changes in calcium levels, viral infections, oxidative stress, inflammation, and hypoxia [14]. While moderate levels of ER stress can be beneficial for cells, helping them to adapt to environmental changes and recover from disruptions to ER homeostasis, excessive ER stress can lead to caspase-12-dependent apoptosis. This process can contribute to the development of several diseases [15]. Studies have shown that ER stress is a significant factor in the development and progression of various disorders, including diabetes, obesity, cancer, inflammation, neurodegenerative diseases, and autoimmune diseases [16].

ER stress in hepatocytes has been implicated in the development of nonalcoholic steatohepatitis (NASH). Tauroursodeoxycholic acid, an ER stress inhibitor, is effective in inhibiting hepatocyte balloon degeneration, apoptosis, and inflammasome activation in obese mice with severe steatosis that is stimulated with lipopolysaccharides (LPS). Additionally, in obese mice, the administration of LPS or tunicamycin (an ER stress inducer) induced NLRP3 inflammasome activation, which subsequently triggered caspase-1, caspase-11, interleukin-1β-mediated hepatocyte pyroptosis, and caspase-3-dependent apoptosis, all of which are key contributors to the development of NASH [17].

ER stress and mitochondrial dysfunction

The term mitochondrial dysfunction refers to diminished mitochondrial biogenesis, altered membrane potential, decreased mitochondrial number, and altered activities of oxidative proteins owing to the accumulation of reactive oxygen species (ROS) in cells and tissues [18]. The proper functioning of mitochondria relies on calcium homeostasis, which necessitates effective calcium signaling to maintain the balance between mitochondrial function and dysfunction, ultimately affecting cell survival [19]. Notably, mitochondria do not produce calcium. Instead, transferring an appropriate amount of calcium from ER to mitochondria is necessary to induce ATP production in the cell. However, excess calcium accumulation in mitochondria triggers the mitochondrial apoptosis pathway [20].

The liver relies on the ER and mitochondria for lipid synthesis and glycolipid metabolism. The ER acts as the primary storage site for calcium and releases it in response to different hormones. When the ER's calcium stores are exhausted, stromal interaction molecules (STIMs) help connect the ER with the plasma membrane, leading to the recruitment and activation of Orai calcium channels [21]. This results in calcium release and activation of the release-activated channel, which increases cytoplasmic calcium levels and triggers downstream signaling. This process is called storage-operated calcium entry. The excess calcium is then pumped back into the ER to replenish the deficiency, thus maintaining a dynamic equilibrium of calcium [8].

Mitochondria-associated ER membranes (MAMs) are contact sites between the ER and mitochondria that facilitate calcium transfer from the ER to mitochondria. This transfer occurs via the formation of a macromolecular complex comprising inositol 1,4,5-trisphosphate receptor (IP3R), glucose-regulated protein 75 (GRP75), and voltage-dependent anion channel 1 (VDAC1) [18]. Liver hyperlipidemia causes an ER membrane lipid and phosphatidylethanolamine imbalance. This leads to elevated cytoplasmic calcium levels and reduced ER calcium levels. Therefore, the dysregulation of calcium transfer from the ER to mitochondria via MAMs can cause mitochondrial calcium overload and mitochondrial dysfunction [22]. In animal models of obesity, IP3R was found to be upregulated and enriched in the mitochondrial-associated membranes. Reducing IP3R expression in animal models can correct mitochondrial dysfunction, which was proved by the fact that animal models with IP3R1 deficiency are protected from lipid deposition in the liver even after high‐fat feeding [23].

ER calcium channels

**Figure 1:**
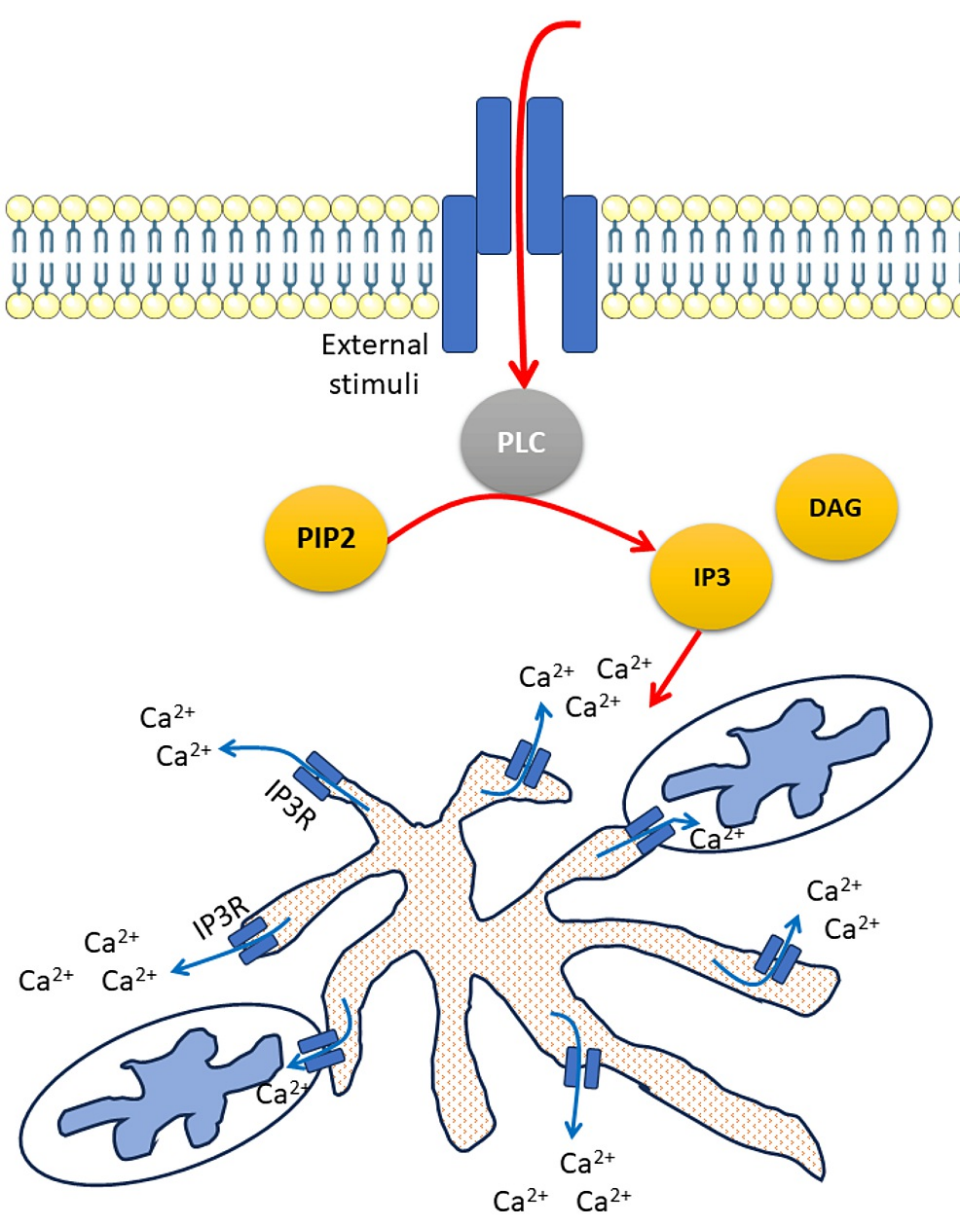
Activation of the endoplasmic reticulum calcium channels Ca: calcium; DAG: diacylglycerol; IP3: inositol triphosphate; PIP2: phosphatidylinositol 4,5-bisphosphate; PLC: phospholipase C This image was created by the authors of this study

Cell activation is complex and involves various extracellular molecules such as hormones, growth factors, and neurotransmitters. When these molecules bind to their respective receptors on the cell surface, it triggers a series of intracellular signaling events that ultimately lead to the activation of phospholipase-C. Once activated, phospholipase-C cleaves a specific type of membrane-bound phospholipid called phosphatidylinositol 4,5-bisphosphate (PIP2) into two-second messengers: diacylglycerol (DAG) and IP3, which then diffuses through the cytoplasm and binds to its tetrameric receptor, IP3R, located on the membrane of ER leading to a conformational change that opens a channel, allowing calcium ions to flow from the ER into the cytoplasm [24] (Figure 1). IP3R is a highly abundant calcium channel found in various tissues throughout the body. It is crucial to consider the subcellular localization of the IP3R and its relevance for various cellular processes as it is localized to the ER membrane, which can be found in many parts of the cell. The calcium signal mediated by IP3R can be fine-tuned by clustering one or more IP3R isoforms in a specific section of the ER membrane. It is the only intracellular calcium-release channel in hepatocytes [25].

**Figure 2:**
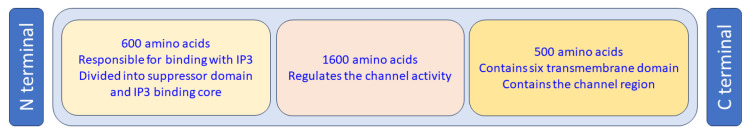
Schematic presentation of the structure of IP3R IP3R: inositol triphosphate receptor This image was created by the authors of this study

IP3R is a large protein with a linear sequence comprising three main sections. The N-terminal section is a region of about 600 amino acids responsible for binding to IP3, a second messenger molecule involved in various cellular processes. This region is further divided into a suppressor domain and an IP3-binding core, with the former preventing the spontaneous opening of the receptor channel [24]. The second section of the IP3R is a modulatory and transducing region that is approximately 1600 amino acids in size. This region plays a crucial role in regulating the channel activity of the IP3R. Finally, the C-terminal section of the IP3R consists of about 500 amino acids and contains six transmembrane domains. This region comprises a channel region and a coupling region, which together form the channel pore (Figure 2). Understanding the structure and function of each section of the IP3R is important for elucidating the molecular mechanisms that underlie IP3-mediated signaling pathways [26].

It consists of three subtypes: IP3R1, IP3R2, and IP3R3, with 2700 amino acids in each subtype. Although these subtypes share similar functions, they differ slightly in their physiological roles. In hepatocytes, IP3R1 and IP3R2 are the predominant isoforms and are both expressed in liver cells, whereas IP3R3 is physiologically deficient. IP3R2 is most concentrated in the region of ER along the canalicular membrane [27]. Although the IP3R isoforms share many similarities, they can vary in their properties. They have different levels of affinity for IP3, with IP3R2 having the highest affinity, followed by IP3R1, then IP3R3. These differences are primarily due to variations in their suppressor domains [28]. The presence of multiple IP3R isoforms holds significant physiological relevance, as they exhibit distinct patterns of expression in diverse subcellular locations across several cell types and organs. Furthermore, these isoforms display altered expression profiles during cellular differentiation and developmental processes and in response to pathological or physiological conditions [29]. This highlights the importance of understanding the functional implications of each IP3R isoform in distinct cellular and physiological contexts.

After IP3R activation by IP3, releasing an excessive amount of calcium contributes to the apoptosis of cells. Notably, increased IP3R activity leads to mitochondrial calcium overload, which activates pro-apoptotic factors. Conversely, low levels of calcium in ER protect against apoptosis. If the levels of calcium in the ER fall too low, it leads to UPR, which is a cellular process that helps mitigate ER stress and ensures proper protein folding [30]. Nuclear IP3P-mediated calcium signals are distinct from cytosolic signals and are important for cell proliferation and liver regeneration. Furthermore, mitochondrial calcium is a key component of cellular metabolism, including in hepatocytes, and regulates apoptosis in the liver [31]. IP3R is crucial for maintaining lipid homeostasis - Drosophila with IP3R mutations tend to become obese and store excess triglycerides in their fat bodies even on a normal diet [32]. While IP3R1 seems to be more important than IP3R2 in regulating mammalian hepatic lipid metabolism, as it selectively couples to mitochondria and regulates lipid droplet formation, hepatic IP3R2 expression is found to be decreased in obese mice [33].

IP3R1 and NAFLD

IP3R1 is a protein that contains several sites for protein kinases and phosphatases to phosphorylate it. Studies indicate that phosphorylation of IP3R1 helps regulate the release of calcium. Various protein kinases can directly phosphorylate IP3R1, and each kinase acts distinctly. For instance, IP3R1 can be phosphorylated by PKA at the Ser1588 or Ser1755 site, which enhances the open probability of IP3R1 [34]. Conversely, PKB phosphorylates IP3R1 at Ser2681, leading to the inhibition of ER calcium release and apoptosis in different cell types [35]. According to recent research, the expression of IP3R1 is one of the contributing factors leading to mitochondrial dysfunction and metabolic imbalance, thus increasing the likelihood of developing NAFLD. On the other hand, inhibiting IP3R1 can protect mitochondrial function and prevent the progression of NAFLD. In addition, liver-specific IP3R1 knockout reduces hepatic triglycerides and makes the mice resistant to NASH [8]. In summary, the IP3R1 signaling pathway regulates mitochondrial function and NAFLD progression.

IP3R2 and NAFLD

IP3R2 has been cited as a critical mediator of hormonal regulation of hepatic glucose production in fasting and diabetes. In the context of obesity, perturbed glucose homeostasis is widely observed. In the process of bile solute secretion, IP3R2 plays a crucial role. Research findings have revealed that during fasting, the levels of IP3R2 are elevated, indicating its importance in regulating bile secretion. Bile salts and organic anion glutathione are essential for the formation of canalicular bile, which helps in the absorption of dietary lipids. A negative feedback loop exists where fatty liver reduces the expression of IP3R2, which hampers the function of the bile salt export pump, leading to a decline in the absorption of additional dietary lipids [36].

The absence of the IP3R2 protein in cells was found to significantly reduce nuclear calcium signaling in comparison to normal cells. This indicates that the activation of the inositol IP3R2 channel is crucial for nuclear calcium signaling. Calcium plays a vital role in the cytoplasm, especially in cell proliferation [37]. Further research has shown that IP3R2-deficient livers experienced significant diffuse steatosis and impaired hepatocyte regeneration after hepatectomy. These findings suggest that higher levels of c-Jun expression negatively impact IP3R2 expression in the livers of individuals with NAFLD. This could potentially lead to impaired liver regeneration and cell proliferation, which ultimately contributes to the progression of NAFLD [7]. Although more research is needed to understand the exact underlying mechanism, it is hypothesized that factors that affect IP3R2 expression could have clinical significance in the treatment of NAFLD.

## Conclusions

NAFLD is a growing healthcare concern worldwide, and it is strongly linked to insulin resistance and obesity. Calcium channels, particularly IP3R, play a critical role in liver development and defense against NAFLD. Calcium dysregulation is a significant risk factor for NAFLD, influencing insulin resistance and glucose intolerance. The two subtypes of IP3R affect NAFLD differently, with IP3R1 deletion impeding development and IP3R2 deletion promoting progression. This information is valuable in identifying potential therapeutic targets for NAFLD and other liver conditions.
